# Assessment of the Pollution Status of Eleyele Lake, Ibadan, Oyo State, Nigeria

**DOI:** 10.5696/2156-9614-7.15.51

**Published:** 2017-09-07

**Authors:** Oluwafunmilayo O. Olayinka, Hakeem Oludare Adedeji, Adeolu Akanji Akinyemi, Olusola Juwon Oresanya

**Affiliations:** 1 Department of Environmental Management and Toxicology, Federal University of Agriculture, Abeokuta; 2 Department of Aquaculture and Fisheries Management, Federal University of Agriculture, Abeokuta

**Keywords:** heavy metals, industrialization, PCBs, pollution, risk assessment, wetland

## Abstract

**Background.:**

Lakes are a vital water resource, but are adversely affected by pollutants such as heavy metals and polychlorinated biphenyls (PCBs) from urban, agricultural and industrial activities. This can give rise to potential pollution-related health problems such as cancer and infectious diseases. Risk assessments are necessary to determine the degree of pollution and its effects on human health and ecological systems.

**Objectives.:**

This study assessed the pollution status and a risk assessment was calculated to determine the degree of the pollution and its effects on the human health and the ecological system of Eleyele Lake in Ibadan, Nigeria.

**Methods.:**

Physical and chemical parameters, heavy metals and PCBs were determined in the lake water using standard methods from December 2013 to February 2014 at ten different sites of anthropogenic activity.

**Results.:**

Water pH ranged from 6.00–7.50, while electrical conductivity ranged from 205.00–221.00 μs/cm^3^. Dissolved oxygen ranged from 0.30–6.00 mg/L and total dissolved solids ranged from 105.00–113.00 mg/L. Phosphate levels ranged from 13–0.99 mg/L. Nitrate and sulphate in the dry season ranged from (3.10–3.80 and 35.81–40.97 mg/L) and (0.12–0.37 and 6.10–10.30 mg/L) in the wet season. Heavy metal concentrations were in the order cadmium (Cd) > zinc (Zn) > copper (Cu) > chromium (Cr) > lead (Pb) for the dry season and Cd > Zn > Cr > Pb > Cu for the wet season. Total PCBs ranged from 493.90–732.55 μg/L and 52.00–390.03 μg/L for the dry and wet seasons, respectively. All determined physical and chemical parameters were within permissible levels, while heavy metals and PCB concentrations were higher than permissible levels.

**Discussion.:**

The hazard quotients and carcinogenic risk values were greater than acceptable limits, indicating that PCBs in Eleyele lake water pose adverse health effects to the local population. It was observed in this study that lower chlorinated PCBs were more prevalent than higher chlorinated PCBs. This may be attributed to the fact that the lower chlorinated PCBs are influenced by atmospheric deposition as a result of their volatility, and they are more susceptible to atmospheric transport than highly chlorinated PCBs.

**Conclusions.:**

PCBs possess serious health risks to the population that depends on the lake as a source of domestic water and its aquatic organisms. Efforts are needed to reduce anthropogenic influence on the lake through strict environmental controls.

## Introduction

The availability of safe and reliable sources of water is an essential prerequisite for development.^1^ However, rapid urbanization, population and industrial growth, and other anthropogenic activities have resulted in the mass generation of domestic, municipal and industrial wastes which are often discharged into surrounding water bodies; making the water unfit for human use as well as threatening aquatic biodiversity. Contamination of fresh water sources such as urban lakes with a wide range of pollutants has become a matter of concern due to the impact on the ecological balance of the recipient environment and its diversity of aquatic organisms.^2^ Eleyele Lake, located in Ibadan, Nigeria, was constructed for the purpose of providing pipe-borne water to the population. It is also used for artisanal fishing and aquaculture activities, irrigation of farmland, domestic uses, and recreation. A good percentage of the domestic fish supply in Nigeria comes from inland waters such as Eleyele Lake. In addition, the lake provides a livelihood to fishermen and others in the supply value chain. However, the sustainability of the fish supply from this lake is threatened by the high density of residential and commercial buildings, intensive agricultural activities and high vehicular traffic within the catchment area. The lake is exposed to various forms of pollutants, including heavy metals and polychlorinated biphenyls (PCBs) from anthropogenic activities such as agriculture and unregulated discharge of industrial wastes. Heavy metals and PCBs are highly mutagenic and carcinogenic and have been recognized as major causes of environmental and health problems such as reproductive, neurological and endocrinal defects in fetal and infant development.^3^,^4^ Increasing urbanization, population growth and associated land use and management changes have resulted in the alteration of the hydrology and water chemistry of Eleyele Lake, thereby causing the water quality to deteriorate. In 2014, Akinyemi et al reported that physico-chemical properties and concentrations of heavy metals in Eleyele Lake are within the permissible limits of the World Health Organization (WHO), but the river water needs treatment to meet the WHO standard limit for potable water.^5^ There is need for regular monitoring and evaluation of the status of the water quality in view of the importance of the lake as a source of water supply for the Ibadan metropolis. The objectives of this study include examination of the physical, chemical and heavy metal concentrations of water from the lake, assessment of the concentration of PCBs in water from the lake and calculation of non-carcinogenic and carcinogenic risks for PCBs.

**Abbreviations**
*BOD*: Biochemical oxygen demand
*COD*: Chemical oxygen demand
*DCM*: Dichloromethane
*DO*: Dissolved oxygen
*EC*: Electrical conductivity
*HQ*: Hazard quotient
*USEPA*: United States Environmental Protection Agency
*WHO*: World Health Organization

## Methods

### Study Area

**Figure 1:**
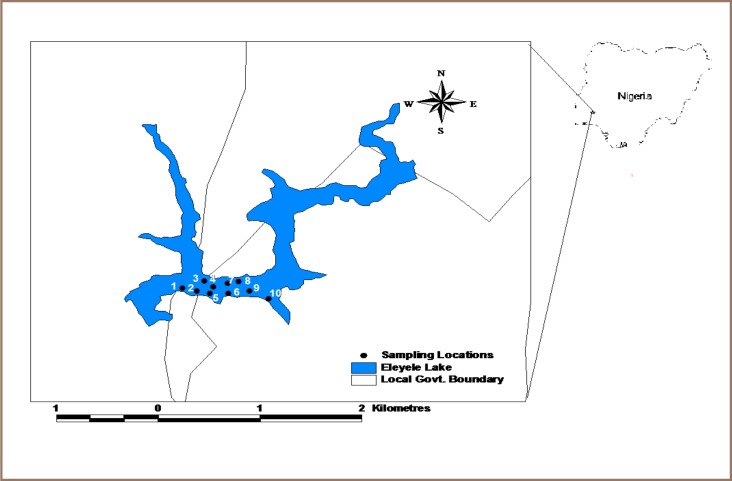
Map of the study area showing sampling points

This study was carried out in Eleyele Lake (*Figure 1*) located in the northeastern part of Ibadan city, southwestern Nigeria within longitude 3° 25′ E– 3° 35′ E and latitude 7° 23′ N– 7° 31′ N. The artificial lake was constructed in 1942 to provide raw water to provide a potable water supply for major parts of Ibadan metropolis. The lake and associated dams at Eleyele receive water from the River Alapata and the headstream of River Ona. The lake has a length of 240 m across the dam, catchments area of 323.7 sq km, and an impoundment area of 156.2 hectares with a storage capacity of 29.5 million litres of water.^6^ The lake is 125 m above sea level with an average depth of 6.0 m. The Eleyele catchment falls within an area that receives a mean annual rainfall of 1981.2 mm and has a mean annual temperature of 28.8°C. The drainage system is controlled by the bedrock geology, with characteristic dendritic pattern of structurally controlled streams and rivulets. Flash overland flow and drainage discharge is common during the wet season from May through October aided by the hilly nature of the surrounding terrain.^6^

Both the quality and quantity of the water in the lake are adversely affected by the expansion in population density around the lake area. The consequent increase in the building density around the reservoir enlarges the impervious area as well as modifies the natural drainage system due to increased paving in the area.^7^ Apart from providing Ibadan megacity dwellers with pipe-borne water, fishing activities are also carried out on the lake. In the past three decades, rapid expansion of the city due to population growth has caused encroachment on the surrounding forest reserve and reduction in the perimeter protection of the lake.^8^ Commercial fishing is also being carried out at Eleyele Lake. Effluents from industries, human wastes, run-off from agriculture farms, automobile workshops and oil from transformers are directly discharged into the river channel that empties into the lake which pollutes the water with PCBs and heavy metals.^9^ Nigeria does not produce PCBs, but contamination arises from importation of electrical transformer oils containing PCBs from developed countries such as France, the United Kingdom and Japan.^9^ Between the 1970s and 1980s, these transformers were widely used in the energy production sector, resulting in PCB and oil leakage into soil and groundwater.^10^ Polychlorinated biphenyls and heavy metals have the potential to bioaccumulate across the food chain, building up in top predators through consumption of contaminated water and biota.

### Sample Collection

Triplicate samples of water from the lake were collected during the day (morning) in each month at ten anthropogenic sites from December 2013 to February 2014, representing the dry season, and from June to August 2014, representing the rainy season. Activities common at the different locations were sample 1] commuter landing site, sample 2] paint factory, sample 3] bathing and baptism, sample 4] smoking of fish, sample 5] automobile workshop, sample 6] palm oil refinery, sample 7] Cassava (*garri*) processing, sample 8] car washing station, sample 9] block making factory and sample 10] plastic industry near a transformer situated upriver of the lake. Samples were collected using pre-washed plastic bottles rinsed with lake water at each sampling point in order to condition the bottle with the lake water sample and then dipped 15 cm below the water level at the designated sampling sites. Samples were then stored in an ice chest. Samples collected for heavy metal analyses were fixed in the field with 3 ml of analar grade nitric acid per 1 L sample while samples for PCB analyses were collected in amber bottles.

### Sample Preparation

Water samples were stored at 4°C in the fridge prior to analysis. Physical and chemical parameters of water such as temperature, pH, and electrical conductivity (EC) were measured *in situ* using a Hanna portable meter, and dissolved oxygen (DO) was also measured *in situ* using a DO meter.^11^ Chemical oxygen demand (COD) was determined using a strong oxidizing agent potassium dichromate and sulphuric acid at 148°C with back titration. Total dissolved solids and total suspended solids were determined gravimetrically.^11^ Nitrate, phosphate and sulphate were determined using a ultraviolet-vis spectrophotometer.^12^

### Sample Digestion

Water samples were digested following the method of the American Public Health Association et al.^13^ Exactly 50 ml of the water sample, 5 ml of concentrated nitric acid along with Hengar granules (anti-bumping agents) were added into a digestion beaker and slowly heated on a hot plate. Evaporation was discontinued just before precipitation occurred. Replenishing of the content was done with 1:1 (vol/vol) nitric acid: perchloric acid mixture until complete digestion was determined, i.e. when a light-coloured clear solution was shown. The digested sample was cooled down, filtered through Whatman paper and quantitatively transferred into a 25 ml volumetric flask and made up to the mark with distilled water. A blank determination was concurrently done. All samples and blanks were stored in plastic containers. Concentrations of heavy metals (copper (Cu), lead (Pb), chromium (Cr), cadmium (Cd), and zinc (Zn)) were determined in each sample solution using a Perkin-Elmer Analyst 300 atomic absorption spectrophotometer. Stock standard solutions for the atomic absorption analyses were obtained from commercial British Drug House metal standards for atomic absorption spectroscopy. Working standard solutions were made from the stock by dilution of measured aliquots with distilled water. Stock solutions of 1000 ppm were prepared by dissolving sulphate salts of copper, lead, chromium, cadmium and zinc for each metal. For standard working solutions, 100 ppm were prepared by pipette in 10 ml of stock standard solution of each metal into different 100 ml volumetric flasks and diluted with distilled water and then thoroughly mixed.

### Extraction Procedure for PCBs in Water

The PCB extraction was based on the United States Environmental Protection Agency (USEPA) standard operating procedures (SW846/3510C/).^14^ Exactly 200 ml of the water sample (unfiltered) was poured into a 500 ml separatory funnel. Then 40 ml of dichloromethane (DCM) was added to it and extracted by shaking the separatory funnel for two minutes, with periodic venting to release excess pressure. The organic layer (generally the bottom layer) was allowed to separate from the water phase for 10 minutes. A second 40 ml portion of DCM was added to the separatory funnel and the extraction procedure was repeated a second time to ensure complete extraction. The combined extract (bottom layer) was filtered through a funnel containing glass wool and 1 g of anhydrous sodium sulphate into a 500 ml Erlenmeyer flask and then washed with 10 ml of DCM for quantitative transfer. The combined sample extracts were evaporated under vacuum using a rotary evaporator at 30°C–35°C to 5 or 6 ml. The concentrated extract was dissolved in 40 ml of n-hexane and evaporated to 1 ml under a gentle stream of nitrogen at 50 to 60°C (water bath) at atmospheric pressure. The extract was solvent exchanged to n-hexane.

### Sample Clean-up

A 600 mm × 19 mm cleanup column was prepared by blocking the hole with glass wool and adding 3 g of activated silica gel (60 mesh), calcined at 450°C for 4 hours, and then stored at 120°C until use. The column was topped with preheated sodium sulfate (previously heated at 650°C for 18 hours), and stored in a clean bottle in a dessicator. The column was rinsed by eluting with 20 ml hexane twice and discarded. The concentrated extract in n-hexane was transferred to the column and eluted with 50 ml of 25 DCM/hexane (vol/vol ratio). The eluate was collected in a 100 ml-round bottom flask. This fraction contained PCBs.^15^ The eluate was reduced by volume with rotary evaporator to 3 ml; the volume was further reduced to 1 ml in a gentle stream of nitrogen and solvent exchanged to n-hexane.^14^ Analysis was conducted using an Agilent model 6890 gas chromatograph coupled with a 63-nickel electron capture detector HP-5MS. All the instrumental conditions were reported in Khaled et al.^16^ Chromatographic separation was achieved on a HP-5MS (Agilent, Folsom, CA) capillary column of (30 m × 0.25 mm × 0.25 μm).

### Quality Assurance and Quality Control

The individual reference standards used for quantification and identification of PCB congeners were obtained from Dr. Ehrenstorfer GmbH (Augsburg, Germany). All reagents used during the analyses were exposed to identical extraction procedures and subsequently run to check for interfering substances. A sample in each series was analyzed in triplicates. The limits of detection of PCBs were defined as five times the signal-tonoise ratio. The recoveries of PCB congeners varied between 80 and 98%.

### Risk Assessment

Risk assessment of the Eleyele Lake water was assessed using the hazard quotient (HQ) and cancer risk assessment. HQ was calculated according to USEPA guidelines using the following equations:

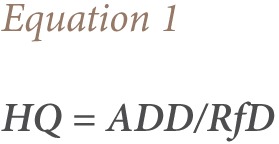



ADD is the intake exposure level (mgkg^−1^day^−1^), and the reference doses are consistent (2 × 10^−5^ mgkg^−1^day^−1^) and calculated by the USEPA for Aroclor 1254.^17^

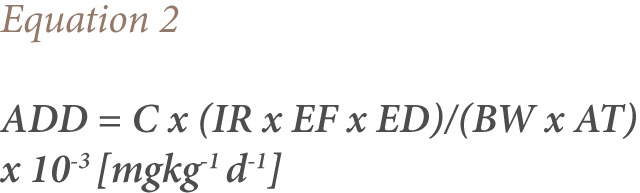
Note: Conversion factor = 10^−3^ × μgL^−1^ to mgL^−1^

Where C represents the average concentration of PCBs during the monitoring period (microgram per litre). For this study, IR represented the daily water intake rates in relation to the age groups according to the Exposure Factors Sourcebook for the European Population which was as follows: 0.3 L day^−1^ for ages 0–6; 1 L day^−1^ for ages 7–17; and 2 L day^−1^ for adults.^18^ The exposure frequency (EF) was 365 days/year. The exposure duration (ED) varied by age group. It was 6 years for ages 0–6, 11 years for ages 7–17 and 30 years for adults. The average body weight was 15 kg for ages 0–6, 46 kg for ages 7–17, and 70 kg for adults.^19^ Averaging time (AT) was ED × 365 days. AT_0–6_ was 2190, AT_7–17_ was 4015 and AT_Adult_ was 10950 days.


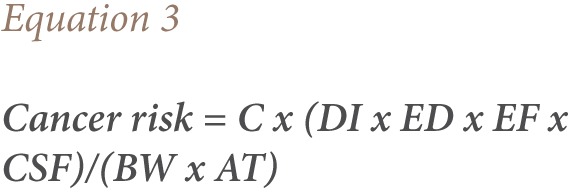


The cancer risk assessment of PCB congeners via water consumption was calculated according to the risk guidelines of the USEPA (*Equation 3*), where C was the concentration of PCBs in the water sample (mg L^−1^); DI is the daily input of 2 L day^−1^; ED was 30 years; body weight was 60 kg; average life span was 70 years × 365 days =25500 days; EF was 365 days/year; cancer slope factor (CSF) was 0.07 (mgkg^−1^day^−1^) for low risk and persistent PCBs according to the USEPA.^20^,^21^

### Statistical Analysis

Results obtained from all samples were subjected to descriptive (mean and standard deviation) and inferential analysis of variance statistics and P<0.05 was considered to indicate statistical significance. Means were separated using Duncan's multiple range test.

## Results

### Physical and Chemical Parameters of Water

In all water samples, the highest pH value recorded was 7.50 during wet season, and the lowest pH value was 6.00 (*Tables 1 and 2*). Mean pH values obtained during the dry season ranged from 6.00 ± 0.29 – 6.90 ± 0.02, and (6.70 ± 0.20 – 7.50 ± 0.20) for the wet season. The lowest (27.30 ± 0.30° C) water temperature was recorded during the wet season, while the highest mean value (27.80 ± 0.02° C) was recorded during the dry season. Values for EC ranged from 205.00 ± 3.50– 217.00 ± 4.60 μs/cm^3^ and 205.00 ± 8.08–221.00 ± 6.93 μs/cm^3^ during the dry and wet seasons, respectively. Nitrate values in water ranged from 3.10 ± 0.07–3.80 ± 0.01 mgL^−1^ and 0.12 ± 0.02 –0.37 ± 0.02 mgL^−1^ during the dry and wet seasons, respectively, showing a considerable rise in the level of nitrate during the dry season when compared to the wet season. There was seasonal variation in phosphate values ranging from 0.13 ± 0.05–0.31 ± 0.03 mgL^−1^ and 0.80 ± 0.01–0.99 ± 0.06 mgL^−1^ for the wet and dry seasons, respectively. Mean values of sulphate across the sampling points varied between 6.10–10.30 mgL^−1^ and 35.81– 40.97 mgL^−1^ during the wet and dry seasons, respectively. Dissolved oxygen values ranged from 1.00 ± 0.60–6.00 ± 0.30 mgL^−1^ and 0.30 ± 0.02–2.30 ± 0.10 mgL^−1^ for the wet and dry seasons, respectively. The highest and lowest biochemical oxygen demand (BOD) values were recorded during the wet season with a mean value of 2.60 ± 0.40 mgL^−1^ and 0.20 ± 0.00 mgL^−1^, respectively. The COD recorded in this study varied between 3.80 mgL^−1^ to 8.80 mgL^−1^ during the dry season, while no value was recorded during wet season. Total dissolved solid values ranged from 104 ± 2.89–113 ± 1.16 mgL^−1^ during the wet season and 105 ± 2.31–111 ± 1.16 mgL^−1^ during the dry season. Total suspended solid values obtained in this study were (3.3–12.5 mgL^−1^ and 5.6–11.2 mgL^−1^) during the wet and dry seasons, respectively.

**Table 1:** Mean Values of Physical and Chemical Parameters of Water During the Wet Season Across Sampling Points

**Sampling Points**											
**Parameters**	**1**	**2**	**3**	**4**	**5**	**6**	**7**	**8**	**9**	**10**	**WHO**
**pH**	6.70±0.20^e*^	6.90±0.20^d^	7.15±0.40^b^	7.00±0.20^c^	7.10±0.20^b^	7.11±0.40^b^	7.20±0.30^b^	6.70±0.20^e^	6.70±0.20^e^	7.50±0.20^a^	6.5–8.5
**Temp (^0^C)**	27.70±0.60^a^	27.50±0.20^bc^	27.60±0.50^ab^	27.50±0.40^bc^	27.60±0.30^ab^	27.30±0.30^d^	27.40±0.20^cd^	27.60±0.30^ab^	27.60±0.80^ab^	27.70±0.20^a^	-
**EC (μscm^−3^)**	217 ±5.20^b^	214±5.77^e^	209±1.16^g^	216±2.89^c^	206±9.82^i^	221±6.93^a^	215±4.62^d^	205±8.08^j^	213±10.40^f^	207±2.89^h^	-
**NO3^−^ (mgL^−^**^1^**)**	0.15±0.02^f^	0.33±0.01^bc^	0.35±0.01^ab^	0.16±0.01^f^	0.32±0.03^cd^	0.24±0.02^e^	0.12±0.02^g^	0.37±0.02^a^	0.31±0.00^cd^	0.30±0.01^d^	45
**PO4^3−^** **(mgL^−^**^1^**)**	0.27±0.02^ab^	0.20±0.06^ab^	0.33±0.12^a^	0.28±0.02^ab^	0.31±0.03^a^	0.27±0.02^ab^	0.29±0.0^ab^	0.28±0.03^b^	0.13±0.05^c^	0.25±0.05^bc^	< 5
**SO4^2−^** **(mgL^−^**^1^**)**	9.32±0.05^c^	9.52±0.06^c^	6.94±0.12^e^	9.42±0.05^c^	9.84±0.06^b^	9.52±0.11^c^	10.30±0.10^a^	7.40±0.10^d^	6.10±0.11^f^	6.10±0.01^f^	250
**DO (mgL^−^**^1^**)**	5.60±0.30^b^	6.00±0.30^a^	5.20±0.40^c^	3.80±0.90^g^	5.00±0.60^c^	4.00±0.50^f^	4.80±0.50^d^	1.00±0.60^h^	4.60±0.90^e^	5.80±1.10^ab^	-
**BOD(mgL^−^**^1^**)**	0.80±0.30^e^	2.60±0.40^a^	0.60±0.40^ef^	1.80±0.30^c^	1.00±0.60^de^	2.20±1.00^b^	0.40±0.10^f^	0.20±0.00^g^	1.20±0.10^d^	0.40±0.10^f^	2
**COD(mgL^−^**^1^**)**	0.00	0.00	0.00	0.00	0.00	0.00	0.00	0.00	0.00	0.00	-
**TDS (mgL^−^**^1^**)**	111±1.160^b^	109±3.46^d^	107±2.31^e^	110±1.73^c^	105±3.46^g^	104±2.89^h^	110±1.73^c^	113±1.16^a^	109±4.04^d^	106±1.73^f^	1000
**TSS (mgL^−^**^1^**)**	10.20±0.37^b^	9.80±0.02^c^	6.40±0.02^e^	9.50±0.15^d^	4.70±0.64^f^	3.90±0.10^g^	9.60±0.40^c^	12.50±0.46^a^	6.50±0.09^e^	3.30±0.10^h^	< 500

^*^Means with the same superscript along the same row (sampling points) are not significantly (p>0.05) different from each other.

Each value represents the mean of three determinations ± standard deviation.

WHO represents stipulated limits (WHO, 2011)^24^

**Table 2:** Mean Values of Physical and Chemical Parameters of Water During the Dry Season Across Sampling Points

**Sampling Points**											
**Parameters**	**1**	**2**	**3**	**4**	**5**	**6**	**7**	**8**	**9**	**10**	**WHO**
**pH**	6.80±0.02^a*^	6.30±0.06^c^	6.40±0.09^c^	6.10±0.04^de^	6.20±0.07^cd^	6.62±0.04^b^	6.00±0.29^e^	6.90±0.02^a^	6.60±0.02^b^	6.70±0.02^b^	6.5–8.5
**Temp (^0^C)**	27.70±0.12^ab^	27.20±0.02^d^	27.50±0.08^bc^	27.60±0.02^abc^	26.67±0.05^e^	27.80±0.02^a^	27.40±0.02^cd^	27.70±0.02^ab^	27.50±0.08^bc^	27.20±0.13^d^	-
**EC (μscm^−3^)**	212±2.30^d^	208 ±1.70^e^	216 ±3.50^b^	206 ±2.30^g^	217±4.60^a^	214±2.90^c^	216±4.00 ^b^	217±2.3 ^a^	207±2.90^f^	205±3.50^h^	-
**NO3^−^ (mgL^−1^)**	3.20±0.20^cd^	3.50±0.01^b^	3.50±0.10^b^	3.40±0.09^bc^	3.30±0.20^bcd^	3.10±0.07^d^	3.50±0.05^b^	3.80±0.01^a^	3.20±0.03^cd^	3.20±0.04^cd^	45
**PO4^3−^ (mgL^−1^)**	0.85±0.01^cde^	0.86±0.01^cde^	0.83±0.01^de^	0.84±0.01^cde^	0.81±0.02^e^	0.80±0.01^e^	0.89±0.01^cd^	0.90±0.01^bc^	0.99±0.06^a^	0.96±0.01^ab^	< 5
**SO4^2−^ (mgL^−1^)**	39.52±0.16^c^	35.81±0.02^h^	36.12±0.05^g^	39.84±0.06^b^	39.84±0.05^b^	37.26±0.04^f^	38.71±0.02^d^	40±0.02^b^	40.97±0.06^a^	37.58±0.07^e^	250
**DO (mgL^−1^)**	1.80±0.04^c^	2.00±0.04^b^	1.30±0.02^de^	0.80±0.01^f^	1.40±0.04^d^	0.90±0.01^f^	0.60±0.02^g^	0.30±0.02^h^	1.20±0.05^e^	2.30±0.10^a^	-
**BOD (mgL^−1^)**	1.30±0.04^c^	1.20±0.02^cd^	2.10±0.02^b^	0.90±0.06^e^	1.10±0.02^d^	0.50±0.03^f^	1.10±0.12^d^	0.30±0.04^g^	1.20±0.01^cd^	2.30±0.04^a^	2
**COD (mgL^−1^)**	5.50±0.20^f^	4.30±0.20^h^	6.90±0.70^d^	7.20±0.30^c^	5.40±0.80^f^	3.80±0.30^i^	6.30±0.20^e^	8.80±1.20^a^	5.10±0.20^g^	7.60±0.50^b^	**^−^**
**TDS (mgL^−1^)**	108 ±2.30^d^	106 ±1.20^f^	111 ±1.16^a^	109 ±2.89^c^	107±2.31^e^	111±1.16^a^	110±0.58^h^	110±4.04^b^	105±2.31^g^	105±4.04^g^	1000
**TSS (mgL^−1^)**	8.50±0.13^e^	6.70±0.08^g^	11.00±0.25^b^	9.30±0.10^d^	7.80±0.05^f^	5.60±0.25^h^	10.60±0.02^c^	11.20±0.17^a^	5.70±0.05^h^	10.90±0.06^b^	< 500

^*^Means with the same superscript along the same row (sampling points) are not significantly (p>0.05) different from each other.

Each value represents the mean of three determinations ± standard deviation.

WHO represents stipulated limits (WHO, 2011)^24^

Abbreviations: Temp, temperature; NO_3_^−^, nitrate; PO_4_^3−^, phosphate; SO_4_^2−^, sulfate; TSS, total suspended solid.

### Heavy Metals Concentration in Water

Mean concentrations of heavy metals analyzed in water during the wet and dry seasons are shown in Table 3. The average concentrations of Cu were 1.13 ± 0.77 mgL^−1^ and 3.46 ± 2.47 mgL^−1^ for the wet and dry seasons, respectively. Mean concentrations of Zn recorded in this study were 2.22 ± 0.58 mgL^−1^ and 4.09 ± 0.49 mgL^−1^ during the wet and dry seasons, respectively. Concentrations of Cr in the lake water were 2.00 ± 0.93 mgL^−1^ during the wet season and 3.15 ± 0.53 mgL^−1^ during dry season. Mean values of Cd in water samples were 3.19 ± 0.40 mgL^−1^ and 12.02 ± 4.69 mgL^−1^ for the wet and dry seasons, respectively. There were seasonal variations in lead with a mean concentration of 1.78 ± 0.76 mgL^−1^ and 1.91 ± 1.19 mgL^−1^ during the wet and dry seasons, respectively. The concentration of Pb recorded in water was higher during the dry season compared to wet season.

**Table 3:** Mean Concentration of Heavy Metals Determined in Water of Eleyele Lake (mgL^−1^) Across Sampling Points

**Sampling Points**													
**HMs**	**Seasons**	**1**	**2**	**3**	**4**	**5**	**6**	**7**	**8**	**9**	**10**	**Mean**	**WHO**
**Cu**	**Wet**	0.24±0.11^e*^	1.23±0.06^bc^	0.40±0.03^d^	1.30±0.03^b^	1.08±0.07^c^	2.10±0.11^a^	0.12±0.09^e^	0.54±0.12^d^	2.22±0.03^a^	2.60±0.17^a^	**1.13±0.77**	**2**
	**Dry**	0.44±0.03^h^	2.52±0.06^e^	0.80±0.03^g^	3.20±0.17^d^	3.08±0.05^d^	8.06±0.06^a^	2.52±0.19^e^	6.60±0.04^b^	5.88±0.15^c^	1.54±0.11^f^	**3.46±2.47**	

**Zn**	**Wet**	1.40±0.08^f^	1.96±0.09^d^	1.76±0.17^de^	2.60±0.03^bc^	1.68±0.05^e^	3.36±0.13^a^	2.42±0.04^c^	2.64±0.11^b^	2.52±0.21^bc^	1.82±0.11^de^	**2.22±0.58**	-
	**Dry**	3.29±0.13^e^	4.55±0.16^b^	4.09±0.09^c^	3.78±0.14^d^	4.39±0.07^b^	4.84±0.13^a^	4.12±0.15^c^	4.11±0.07^c^	3.36±0.09^e^	4.41±0.09^b^	**4.09±0.49**	

**Cr**	**Wet**	0.58±0.11^h^	2.04±0.11^d^	2.90±0.09^b^	0.90±0.19^g^	2.81±0.07^b^	3.57±0.16^a^	1.54±0.17^e^	1.16±0.05^f^	2.17±0.04^cd^	2.30±0.11^c^	**2.00±0.93**	**0.05**
	**Dry**	2.64±0.13^f^	2.73±0.12^ef^	2.80±0.07^ef^	3.00±0.21^de^	3.64±0.10^b^	4.18±0.05^a^	3.30±0.09^c^	2.52±0.21^f^	3.64±0.32^b^	3.12±0.13^cd^	**3.15±0.53**	

**Cd**	**Wet**	2.55±0.18^d^	3.22±0.08^b^	2.91±0.17^c^	3.05±0.1^bc^	3.01±0.11^bc^	3.71±0.15^a^	3.19±0.05^b^	3.80±0.11^a^	3.60±0.19^a^	2.91±0.18^c^	**3.19±0.40**	**0.003**
	**Dry**	3.36±0.15^j^	13.00±0.21^e^	11.00±0.12^g^	6.80±0.07^i^	17.43±0.11^b^	18.2±0.03^a^	17.16±0.07^c^	11.22±0.09^f^	13.44±0.13^d^	8.58±0.03^h^	**12.02±4.69**	

**Pb**	**Wet**	0.92±0.18^g^	2.17±0.04^c^	1.89±0.16^de^	0.87±0.15^g^	2.67±0.15^b^	3.12±0.15^a^	2.10±0.13^cd^	0.86±0.12^g^	1.73±0.12^e^	1.44±0.19^f^	**1.78±0.76**	**0.01**
	**Dry**	0.28±0.03^i^	0.63±0.06^h^	1.10±0.03^g^	1.40±0.03^f^	3.08±0.03^b^	4.16±0.07^a^	2.86±0.15^c^	2.64±0.13^d^	1.80±0.07^e^	1.12±0.09^g^	**1.91±1.19**	

*Means with the same superscript along the same row are not significantly (p>0.05) different from each other.

Abbreviation: HMs, heavy metals.

Each value represents the mean of three determinations ± standard deviation.

WHO represents stipulated limits (WHO, 2011)^24^

### Concentrations of PCBs in Water

Concentrations of PCBs in water samples during the wet and dry seasons are shown in Tables 4 and 5. PCBs in sampling points 1 (15.60 ± 4.05 μgL^−1^) and 4 (13.97 ± 3.17 μgL^−1^) were higher than the PCBs in other sampling points determined during the wet season while PCBs in sampling points 10 (43.09 ± 4.99 μgL^−1^) and 8 (34.08 ± 3.18 μgL^−1^) were higher than the PCBs in other sampling points during the dry season. The highest (43.09 ± 4.11 μgL^−1^) PCB concentration was recorded during the dry season, while the lowest (2.07 ± 0.42 μgL^−1^) PCB concentration was recorded during the wet season. Total PCBs ranged from 52.00–390.03 μgL^−1^ during the wet season and 493.90–732.55 μgL^−1^ during the dry season.

**Table 4:** Mean Concentration of PCBs in Eleyele Water Samples During the Wet Season (μgL^−1^) Across Sampling Points.

Sampling Points										
PCBs	1	2	3	4	5	6	7	8	9	10
PCB 8	1.29±0.01^b*^	0.19±0.01^fg^	0.28±0.01^e^	1.48±0.00^a^	0.06±0.00	0.36±0.35^d^	0.22±0.02^f^	0.21±0.01^f^	0.16±0.01^g^	0.62±0.01^c^
PCB 18	8.25±0.03^b^	2.37±0.02^g^	4.44±0.07^d^	10.48±0.02^a^	1.59±0.21^h^	1.39±0.02^h^	3.88±0.02^e^	3.41±0.01^f^	2.51±0.01^g^	6.58±0.01^c^
PCB 28	4.46±0.02^b^	1.31±0.01^g^	2.20±0.06^d^	5.62±0.07^a^	1.59±0.04^f^	1.37±0.01^g^	1.97±0.02^e^	2.19±0.01^d^	1.89±0.01^e^	2.91±0.01^c^
PCB 44	4.93±0.02^b^	0.95±0.03^i^	1.94±0.01^d^	5.59±0.08^a^	1.84±0.01^e^	1.25±0.02^h^	1.59±0.02^f^	2.03±0.01^d^	1.35±0.02^g^	2.72±0.01^c^
PCB 52	9.37±0.01^b^	1.30±0.12^i^	3.79±0.02^e^	10.87±0.05^a^	3.79±0.01^e^	1.80±0.01^h^	3.19±0.01^f^	4.23±0.01^d^	2.51±0.01^g^	5.09±0.01^c^
PCB 66	11.12±0.01^b^	2.09±0.04^h^	4.10±0.06^e^	16.24±0.10^a^	4.50±0.23^d^	2.96±0.16^g^	3.46±0.02^f^	4.84±0.01^c^	4.37±0.01^de^	3.59±0.01^f^
PCB 77	10.84±0.02^a^	0.85±0.02^h^	1.69±0.04^ef^	7.66±0.17^b^	3.71±0.01^c^	1.09±0.02^g^	1.54±0.05^f^	2.48±0.01^d^	1.73±0.01^e^	2.59±0.01^d^
PCB 81	33.08±0.02^a^	5.23±0.02^i^	10.51±0.06^f^	31.93±0.31^b^	10.93±0.31^e^	7.56±0.03^h^	9.53±0.05^g^	12.31±0.01^d^	11.25±0.01^e^	17.73±0.01^c^
PCB 101	52.59±0.01^a^	7.15±0.03^j^	17.82±0.17^g^	48.92±0.36^b^	22.29±0.36^e^	11.56±0.05^i^	15.13±0.01^h^	23.15±0.02^d^	20.54±0.01^f^	24.29±0.01^c^
PCB 105	18.78±0.01^a^	1.43±0.02^h^	6.45±0.04^e^	15.59±0.21^b^	6.86±0.10^d^	4.58±0.02^g^	5.71±0.02^f^	6.86±0.01^d^	6.72±0.02^d^	8.81±0.01^c^
PCB 114	9.31±0.01^b^	1.46±0.02^g^	3.59±0.10^d^	9.50±0.02^a^	3.64±0.10^d^	2.11±0.01^f^	2.95±0.01^e^	3.72±0.01^d^	3.65±0.01^d^	4.55±0.01^c^
PCB 118	0.06±0.00	0.72±0.173^a^	0.06±0.00	0.06±0.00	0.06±0.00	0.06±0.00	0.06±0.00	0.06±0.00	0.06±0.00	0.06±0.00
PCB 126	47.79±0.01^a^	1.81±0.06^j^	15.97±0.03^g^	40.00±0.06^b^	18.05±0.15^d^	11.03±0.01^i^	14.35±0.02^h^	16.98±0.01^e^	16.55±0.01^f^	22.00±0.29^c^
PCB 128	40.10±0.01^a^	5.89±0.03^i^	14.59±0.04^f^	35.22±0.06^b^	16.72±0.26^d^	8.89±0.01^h^	12.41±0.01^g^	14.67±0.01^f^	15.21±0.01^e^	19.96±0.01^c^
PCB 138	0.07±0.00	0.07±0.00	0.07±0.00	0.07±0.00	0.07±0.00	0.07±0.00	0.07±0.00	0.07±0.00	0.07±0.00	0.07±0.00
PCB 153	0.09±0.00	0.29±0.02^a^	0.09±0.00	0.09±0.00	0.09±0.00	0.09±0.00	0.09±0.00	0.09±0.00	0.09±0.00	0.09±0.00
PCB 156	0.06±0.00	0.06±0.00	0.06±0.00	47.19±0.42^a^	18.59±0.16^c^	12.22±0.01^d^	0.06±0.00	20.22±0.01^b^	0.06±0.00	0.06±0.00
PCB 157	67.55±0.03^a^	5.38±0.04^f^	21.97±0.03^d^	0.08±0.00	0.08±0.00	0.08±0.00	20.10±0.06^e^	0.08±0.00	22.07±0.01^c^	31.28±0.01^b^
PCB 167	0.03±0.00	2.17±0.02^a^	0.03±0.00	0.03±0.00	0.03±0.00	0.03±0.00	0.03±0.00	0.03±0.00	0.03±0.00	0.03±0.00
PCB 169	0.08±0.00	2.66±0.06^h^	5.82±0.02^c^	11.12±0.23^a^	5.53±0.10^d^	3.48±0.02^g^	4.03±0.01^f^	4.37±0.01^e^	5.33±0.01^d^	6.60±0.02^b^
PCB 170	0.03±0.00	0.03±0.00	0.03±0.00	0.03±0.00	0.03±0.00	0.03±0.00	0.03±0.00	0.03±0.00	0.03±0.00	0.03±0.00
PCB 180	48.64±0.08^a^	5.65±0.01^h^	13.60±0.06^de^	33.27±0.26^b^	13.66±0.06^de^	8.40±0.03^g^	12.46±0.01^f^	13.85±0.01^d^	13.56±0.02^e^	18.98±0.01^c^
PCB 187	0.03±0.00	0.68±0.02^e^	6.54±0.10^a^	0.03±0.00	0.03±0.00	0.03±0.00	1.31±0.01^d^	1.47±0.01^c^	1.29±0.01^d^	2.23±0.01^b^
PCB 195	3.16±0.02^b^	0.47±0.01^g^	1.18±0.02^d^	2.81±0.36^c^	7.16±0.26^a^	0.13±0.00	0.91±0.01^e^	0.72±0.01^f^	0.67±0.01^f^	0.13±0.00
PCB 206	18.33±0.04^a^	1.78±0.01^i^	5.60±0.05^d^	15.28±0.04^b^	3.70±0.01^f^	1.97±0.01^h^	5.17±0.01^e^	2.04±0.01^h^	3.61±0.01^g^	7.88±0.02^c^

Total PCBs	390.03	52.00	142.44	349.15	144.62	82.55	120.24	140.10	135.29	188.89
Mean	15.60±4.05	2.08±0.42	5.70±1.28	13.97±3.17	5.79±1.37	3.30±0.82	4.81±1.15	5.60±1.39	5.41±1.38	7.56±1.83

^*^Means with the same superscript along the same row are not significantly (p>0.05) different from each other.

Each value represents the mean of three determinations ± standard deviation.

WHO represents stipulated limits (WHO, 2011)^24^

**Table 5:** Mean Concentration of PCBs in Eleyele Water Samples During the Dry Season (μgL^−1^) Across Sampling Points

Sampling Points										
PCBs	1	2	3	4	5	6	7	8	9	10
PCB 8	10.70±0.02^j*^	26.45±0.08^g^	31.55±0.16^e^	32.55±0.05^d^	46.40±0.02^a^	11.10±0.12^i^	24.90±0.24^h^	38.65±0.13^b^	34.45±0.58^c^	28.60±0.58^f^
PCB 18	11.60±0.61^j^	35.10±1.48^d^	49.10±0.49^c^	49.85±0.73^b^	24.95±0.38^g^	26.95±0.20^f^	21.45±0.39^h^	64.70±0.83^a^	34.80±0.48^e^	19.35±0.13^f^
PCB 28	38.15±0.32^b^	28.90±0.21^e^	32.45±0.39^d^	29.75±0.46^e^	24.65±0.07^g^	26.40±0.69^f^	16.75±0.48^i^	36.60±0.49^c^	19.65±0.07^h^	59.65±0.53^a^
PCB 44	37.20±0.34^b^	23.65±0.49^g^	31.30±0.76^d^	26.90±0.02^f^	23.60±0.02^g^	29.30±0.29^e^	16.60±0.1^h^	34.45±0.06^c^	24.60±0.04^g^	64.35±0.62^a^
PCB 52	16.10±0.25^j^	35.55±0.15^e^	53.35±1.34^b^	42.90±0.13^d^	29.10±0.22^g^	48.95±0.73^c^	21.45±0.08^h^	53.95±0.16^a^	29.55±0.08^f^	19.45±0.05^i^
PCB 66	41.40±0.05^f^	55.45±0.13^d^	59.45±0.53^b^	44.60±0.13^e^	39.60±0.51^g^	56.10±0.94^c^	27.30±0.15^i^	61.50±0.18^a^	31.45±0.51^h^	22.20±0.19^j^
PCB 77	38.45±0.16^b^	28.35±0.13^e^	30.85±0.15^d^	25.35±0.55^f^	21.40±0.75^g^	48.20±0.41^a^	18.75±0.09^h^	33.35±0.14^c^	19.65±0.09^h^	21.85±0.07^g^
PCB 81	37.70±0.42^c^	25.60±0.53^e^	23.40±0.55^f^	19.60±0.04^h^	18.65±0.49^i^	21.90±0.48^g^	42.90±2.84^b^	26.60±0.55^d^	16.90±0.54^j^	44.15±0.36^a^
PCB 101	34.65±1.09^c^	30.55±2.62^e^	34.20±0.66^cd^	26.20±0.27^f^	22.60±2.37^g^	33.35±1.63^d^	59.45±2.62^b^	34.45±0.62^c^	22.30±0.25^h^	63.10±2.59^a^
PCB 105	19.30±0.31^f^	16.75±0.08^g^	17.95±0.19^g^	16.80±0.12^h^	51.40±0.16^a^	17.95±0.04^g^	26.65±0.50^d^	22.95±0.12^e^	29.05±0.29^c^	29.85±0.75^b^
PCB 114	56.40±0.24^a^	37.85±0.51^e^	48.25±0.36^b^	35.05±0.35^f^	32.10±0.64^g^	47.95±0.19^c^	19.60±0.52^i^	46.95±0.13^d^	24.75±0.79^h^	19.60±0.05^g^
PCB 118	0.06±0.00	0.06±0.00	0.06±0.00	0.06±0.00	0.06±0.00	0.06±0.00	0.06±0.00	0.06±0.00	18.20±0.32^a^	0.06±0.00
PCB 126	32.00±0.61^c^	27.00±0.54^e^	18.40±0.29^h^	19.45±0.10^g^	22.10±0.34^f^	29.15±0.22^d^	51.10±0.22^b^	26.95±0.71^e^	29.10±0.27^d^	68.10±0.36^a^
PCB 128	29.90±0.66^c^	26.20±0.23^e^	24.65±1.09^f^	23.40±0.17^g^	19.95±0.24^g^	27.80±0.41^d^	46.30±0.28^b^	24.75±1.56^f^	16.95±0.25^h^	62.55±0.33^a^
PCB 138	0.07±0.00	0.07±0.00	0.07±0.00	0.07±0.00	0.07±0.00	0.07±0.00	0.07±0.00	0.07±0.00	0.07±0.00	0.07±0.00
PCB 153	0.09±0.00	0.09±0.00	0.09±0.00	0.09±0.00	0.09±0.00	0.09±0.00	0.09±0.00	0.09±0.00	49.55±0.00^a^	0.09±0.00
PCB 156	0.09±0.00	0.09±0.00	31.30±0.43^b^	0.09±0.00	23.30±0.22^d^	29.65±1.54^c^	62.75±0.09^a^	0.09±0.00	0.09±0.00	0.09±0.00
PCB 157	41.80±0.10^b^	31.15±0.25^d^	0.08±0.00	31.20±0.83^d^	0.08±0.00	0.08±0.00	0.08±0.00	32.95±0.24^c^	16.95±0.25^e^	78.10±1.24^a^
PCB 167	0.03±0.00	0.03±0.00	0.03±0.00	0.03±0.00	0.03±0.00	0.03±0.00	0.03±0.00	0.03±0.00	42.65±0.07^a^	0.03±0.00
PCB 169	16.65±0.08^f^	16.30±0.05^g^	39.65±0.08^c^	51.35±0.04^a^	44.90±0.19^b^	16.65±0.08^f^	24.05±0.04^e^	16.90±0.21^f^	37.75±0.11^d^	0.08±0.00
PCB 170	0.03±0.00	0.03±0.00	0.03±0.00	0.03±0.00	0.03±0.00	0.03±0.00	0.03±0.00	0.03±0.00	0.03±0.00	0.03±0.00
PCB 180	29.05±0.12^c^	27.10±0.25^d^	29.40±0.13^c^	23.45±0.54^f^	19.40±0.08^g^	19.75±0.13^g^	44.35±0.07^b^	24.65±0.22^e^	16.25±0.04^h^	59.65±0.19^a^
PCB 187	22.30±0.12^d^	28.20±0.18^a^	19.60±0.05^e^	24.20±0.19^b^	0.03±0.00	0.03±0.00	0.03±0.00	22.60±0.05^c^	18.15±0.05^f^	0.03±0.00
PCB 195	0.08±0.00	16.60±0.20^f^	18.25±0.06^e^	19.05±0.10^d^	0.08±0.00	18.25±0.06^e^	43.15±0.06^b^	27.80±0.08^c^	67.60±0.18^a^	42.60±0.53^b^
PCB 206	17.90±0.04^h^	46.05±0.10^b^	31.45±0.09^c^	16.75±0.11^i^	29.80±0.08^d^	48.10±0.09^a^	26.85±0.14^g^	16.75±0.11^i^	27.90±0.05^f^	29.40±0.07^e^

Total PCBs	531.25	562.80	624.55	558.40	493.90	557.50	594.40	647.50	628.20	732.55
Mean	29.51±2.94	29.62±2.21	32.87±2.80	29.39±2.48	29.05±2.50	30.97±3.15	33.02±3.55	34.08±3.18	28.56±2.69	43.09±4.99

^*^Means with the same superscript along the same row (sampling points) are not significantly (p>0.05) different from each other.

Each value represents the mean of three determinations ± standard deviation.

WHO represents stipulated limits (WHO, 2011)^24^

## Discussion

The mean values of the physical and chemical properties of water in Eleyele Lake during the dry and wet seasons are presented in Tables 1 and 2. The pH of water samples from the lake varied at each sampling point and mean values of pH across sampling sites were significantly different from each other. Mean pH values obtained during the dry season were slightly acidic and lower than the values obtained for wet season. The low pH obtained during the dry season could be attributed to the increase in respiration from biota which leads to the release of carbon dioxide in the water body, thereby increasing the production of carbonic acid and hydroxyl ions, which in turn increases acidity. Furthermore, the high mean pH reported in this study during the wet season might be due to the inflow of refuse into the river and production of hydroxyl ions during the rainy season. Extremely high or low pH values in surface waters are harmful to aquatic fauna, as this damages fish gills, eyes and skin and it also affects fish production.^22^ The lowest water temperature was recorded during the wet season, while the highest mean surface water temperature was observed during the dry season. The result obtained was similar to the findings of Akinyemi et al. who reported a temperature in Eleyele Lake of 27.60°C.^5^ The temperature observed in this study conformed to the recommended value of 27°C and 28° C for fresh water.^23^ The water temperature of 28.6–32.7°C (av. 31.0°C) for the main lake reflects a tropical lake setting influenced by solar heat exchange.^7^ Electrical conductivity values recorded throughout the study period were within the WHO permissible limit.^24^ Tijani. obtained a similar EC range of 250–344 μs/cm^3^ in the same lake.^7^ Increasing EC in both seasons demonstrated the presence of inorganic ions like hydrogen ion, sodium ion, potassium ion, magnesium ion, calcium ions, chloride, sulfate and bicarbonate in increasing concentrations in the lake. These ions can influence the conductivity of water. There was a considerable rise in nitrate values during the dry season compared to the wet season (*Tables 1 and 2*). Mean concentrations of nitrate in both seasons were, however, below the WHO tolerance limits of 45 mgL^−1^.^24^

Nitrate is an essential nutrient for aquatic plants and seasonal fluctuations can be caused by plant growth and the decay and rise in the concentration of nitrate might lead to eutrophication and methaemoglobinaemia if not properly controlled.^23^ There were seasonal variations in phosphate values for the dry and wet seasons. The phosphate values recorded in this study during both seasons were below the WHO permissible limit of < 5 mgL^−1^.^24^ The increase in the concentration of phosphate in the water may be attributed to the use of phosphate-containing soaps and detergents by car washing stations, people washing clothes along the banks of the lake and other anthropogenic activities. This increase in phosphate values in rivers results in eutrophication. Sulphate values obtained in this study varied between the sampling sites and the seasons. Low values of sulphate in the wet season may be due to dilution since sulphate salts are soluble in water and the increased level of sulphates during the dry season may be due to the lower volume of water in the lake. Sulphate values recorded in this study were below the WHO permissible limit of 250 mgL^−1^.^24^ Sulphate is generally considered to be non-toxic, however, drinking water containing high amounts of magnesium sulphate or sodium sulphate may lead to intestinal discomfort, diarrhea and consequent dehydration, especially in drinking water containing >500 mgL^−1^ of sulphate.^25^

High dissolved oxygen values were recorded during the wet season. This may be due to increased aeration as a result of high humidity and low temperatures during the wet season. The low DO value obtained during dry season in this study may be due to increased respiration and photosynthesis as well as chemical and biological oxidation processes in the water. High DO values may be due to low organic enrichment as well as pollution of the water body. Uyom et al. stated that DO indicates the health of an aquatic environment, and the metabolism of aerobic organisms and respiration depends on the amount of oxygen dissolved in water.^22^ Dissolved oxygen is essential for the survival of aquatic life and it is used to assess the degree of freshness of a river. A DO value as low as 1–5 mgL^−1^ may reduce the growth rate of fishes when continuously exposed, while a value below 1 mgL^−1^ is reported to be fatal to fish when exposed for more than a few hours.^26^ The highest and lowest BOD values were recorded during the wet season and were slightly higher than the WHO acceptable limit of 2.0 mgL^−1^.^24^ This may be due to the consumption of oxygen available in the lake by bacteria which give rise to unpleasant color and odor of the water body. In general, BOD depends on temperature, degree of biochemical activities, concentration of organic matter and other related factors.

No value was recorded for COD during the wet season. This may be attributed to the increase in the amount of rainfall during this season which results in dilution and reduction of pollutants in the water body. The increase observed in COD values recorded during the dry season may be due to an increase in domestic sewage discharged into the lake. The COD recorded in this study was below the acceptable value for unpolluted surface water quality which is 20 mgL^−1^.^27^

Total dissolved solids is the measure of the sum of all organic and inorganic substances in a liquid in molecular, ionized or micro-granular colloidal suspended form.^23^ Udoh et al. reported that large amounts of dissolved solids can result in an increasing level of minerals of the receiving water body with the consequence of dissolved oxygen depletion.^28^ High total dissolved solids (TDS) values recorded during the wet season may be due to an increased influx of dissolved salt as a result of increased rainfall. Total dissolved solids increased significantly with an increase in EC. The TDS values obtained throughout the study period were within WHO permissible limits.^24^ High levels of TDS, especially when due to dissolved salts, may affect different forms of aquatic biota, as salt dehydrates the skin of fishes. Total suspended solids values obtained in this study during the wet and dry seasons were below the level indicative of pollution (<500 mgL^−1^) stated by the WHO.^24^ Increasing levels of total suspended solids observed in this study may be due to the presence of increasing organic and inorganic pollutants sorbed in the soils near the water body. High concentrations of suspended solids may settle at the bottom of the rivers and cover aquatic fauna, eggs or macro–invertebrate larva leading to the death of benthic organisms.

### Heavy Metal Concentrations in Water

Mean concentrations of heavy metals analyzed in water during the wet and dry seasons are shown in Table 3. Eleyele Lake has been subjected to a high amount of anthropogenic pollutants and this has rendered the water unfit for human use. Low levels of Cu during the wet season may be due to washing away of waste containing Cu. Mean concentrations of Cu obtained in this study exceeded the WHO permissible limit of 2 mg/L.^24^ Zinc has been reported to be neurotoxic in high concentrations resulting in neuronal cell death in a dose dependent manner.^29^ Its deficiency may result in several disorders, while excessive intake of Zn may lead to acute adverse effects. Chromium concentrations in both the wet and dry seasons were higher than reported in the findings of Akinyemi et al. (0.026 mgL^−1^) in Eleyele Lake during the wet season.^5^ The mean concentration of Cr was higher than the WHO^23^ permissible limit of 0.05 mgL^−1^. Chromium has been reported to have adverse effects on skin such as ulcerations, dermatitis and other skin lesions, while respiratory symptoms may include coughing, wheezing, shortness of breath and nasal itching as reported by Alani et al.^30^

Cadmium was the most accumulated metal in water in both seasons. Accumulation of Cd in the human body may cause kidney dysfunction, skeletal damage and reproductive deficiency.^24^ Alshikh et al. reported that Cd in large concentrations may affect the digestive, immune and reproductive system of fish.^31^ The mean concentration of Cd was higher than the WHO permissible limit of 0.003 mgL^−1^.^24^ The increased level of Cd observed in this study may be attributed to burning of tires in the study area.

There were seasonal variations in lead during the wet and dry seasons. The mean concentration of Pb obtained in this study was higher than the WHO permissible limit of 0.01 mgL^−1^.^24^ The high levels of Pb obtained in this study may be due to washing away of dust holding large amount of Pb from the combustion of petrol in automobiles from the road and automobile workshops into the lake. Accumulation of metals was in the order Cu<Pb< Cr< Zn< Cd during the wet season and Pb< Cr< Cu< Zn< Cd during the dry season. Generally, the heavy metal concentration was higher during the dry season than the wet season. This may be attributed to a more gentle flow of the river during the dry season coupled with conditions of low dissolved oxygen when temperatures were slightly higher during the dry season in which most of the metal-bearing suspended particles had settled to form part of the sediment. Likewise, water volume was reduced during the dry season, resulting in dissolved metals in higher concentrations in the liquid phase.

Concentrations of PCBs in water samples during the wet and dry seasons are shown in Tables 4 and 5. A total of 25 PCB congeners were detected in water samples. Levels of PCBs in site 1 were much higher than the PCB levels in other sites in the wet season, while PCB levels in sites 10 and 8 were much higher than PCB levels in other sites during the dry season. The highest PCB concentration was recorded during the dry season, while the lowest PCB concentration was recorded during the wet season. It was observed in this study that lower chlorinated PCBs were more prevalent than higher chlorinated PCBs and this may be attributed to the fact that the lower chlorinated PCBs are influenced by atmospheric deposition as a result of their volatility and they are more susceptible to atmospheric transport than highly chlorinated PCBs. In addition, PCBs 126, 128, 157 and 180 were widely distributed in the environment. These persistent, bioaccumulative organic pollutants may cause long-term impacts on wildlife, whole ecosystems, and human health. Elevated levels of PCBs were observed in Eleyele Lake due to direct deposition of waste from small-scale industries close to the lake and other anthropogenic sources. Lubricants from the transformer (transformer oil) cited at the upstream may leach into the lake causing high concentrations of PCBs in the water.

### Risk Assessment

The total PCBs concentrations in all water samples in this study for the wet and dry seasons were 1745.31 μgL^−1^ and 5931.05 μgL^−1^, respectively (*Table 6*). The concentrations in wet and dry seasons were 32 and 110 times higher than those obtained by Megahed et al. in a study of polychlorinated biphenyls pollution in the Nile River, Egypt.^32^ These values were much greater than the USEPA set value of 500 ng L^−1^ for PCBs in drinking water.^33^ Table 6 also shows individual average daily doses and hazard quotients of contaminated water with PCBs. The HQ values for the wet season ranged from 50–743 and 493–1046 for the dry season. The HQ values were greater than 1, which indicates a possibility of noncarcinogenic effects occurring. Thus, a cancer risk assessment of PCBs was calculated in the ten sampling sites along Eleyele Lake for the wet and dry seasons. There were seasonal variations in the cancer risk calculated for all sampling sites. The cancer risk for the wet season ranged from 8.89×10–5–3.49×10–4, while that of the dry season ranged from 5.31×10–4–7.33×10–4. Cancer risk for the dry season were higher than the USEPA acceptable or tolerable risk level for regulatory purposes, which is within the range of 1×10–6–1×10–4.^34^ In the wet season, cancer risks were within the limit at some sampling sites. Generally, cancer risks were lower in the wet season compared to the dry season. Non–cancer hazards were greater than the cancer hazards. Consequently, these results indicated that the Eleyele Lake water is unsafe for human consumption and poses a carcinogenic risk. The results of the physical, chemical, heavy metals and PCB concentration analysis of Eleyele Lake indicate that it is impacted by increasing human activities which affect water quality as well as the quality of the overall ecosystem.

**Table 6:** Individual Average Daily Dose (mgkg^−1^day^−1^) Hazard Quotients of Investigated PCBs Across Age Groups

Water Sampling Site	Total PCBs (μgL**^−^**^1^) Wet season	Total PCBs (μgL**^−^**^1^) Dry season	ADD/HQ _0–6_ Wet Season	ADD/HQ _0–6_ Dry Season	ADD/HQ _7–17_ Wet Season	ADD/HQ _7–17_ Dry Season	ADD/HQ _Adult_ Wet Season	ADD/HQ _Adult_ Dry Season	Cancer Risk Wet Season	Cancer Risk Dry Season
1	390.03	531.25	0.007/**400**	0.011/**531**	0.0008/**42.39**	0.012/**577**	0.011/**557**	0.015/**758**	3.90E-04	5.31E-04
2	52.00	562.80	0.001/**50**	0.011/**562**	0.001/**56.22**	0.012/**611**	0.015/**743**	0.016/**804**	5.20E-05	5.62E-04
3	142.44	624.55	0.003/**150**	0.012/**624**	0.003/**154**	0.014/**678**	0.004/**203**	0.018/**892**	1.42E-04	6.24E-04
4	349.15	558.40	0.007/**350**	0.011/**558**	0.008/**379**	0.012/**606**	0.010/**498**	0.016/**797**	3.49E-04	5.58E-04
5	144.62	493.90	0.003/**150**	0.010/**493**	0.003/**157**	0.011/**536**	0.004/**206**	0.014/**705**	1.44E-04	4.93E-04
6	82.55	557.50	0.002/**100**	0.011/**557**	0.002/**89.73**	0.012/**605**	0.002/**117**	0.016/**796**	8.25E-05	5.58E-04
7	120.24	594.40	0.002/**100**	0.011/**594**	0.003/**130**	0.012/**646**	0.003/**17.17**	0.017/**849**	1.20E-04	5.94E-04
8	140.10	647.50	0.003/**150**	0.012/**647**	0.003/**152**	0.014/**703**	0.004/**200**	0.018/**925**	1.40E-04	6.48E-04
9	135.29	628.20	0.003/**150**	0.012/**628**	0.003/**147**	0.014/**682**	0.004/**193**	0.018/**897**	1.35E-04	6.28E-04
10	188.89	732.55	0.004/**200**	0.014/**732**	0.004/**205**	0.016/**796**	0.005/**269**	0.021/**1046**	8.89E-05	7.32E-04

**Total**	**1745. 31**	**5931.05**								

## Conclusions

The elevated levels of heavy metals and PCBs observed in the water from Eleyele Lake may pose a danger to the local population who consume water and fish from the lake. The HQ indicated that there is a chance of non-carcinogenic effects occurring, while the cancer risk was significantly higher than the acceptable USEPA tolerable risk for regulatory purposes. It is imperative that the Ministry of Environment of the State provide a health advisory for the consumption of water and control the increasing encroachment of farming and building activities around the Eleyele Lake area. We recommend the adoption of an Integrated Water Resources and Environmental Management plan in order to ensure overall quality of the ecosystem in the area and safeguard the health of the people. Additional research should be conducted on affected fish tissues and further studies are needed to assess the effectiveness of drinking water treatment plants that supply drinking water to the public.
